# Functional Conservation and Divergence of Soybean GmSTOP1 Members in Proton and Aluminum Tolerance

**DOI:** 10.3389/fpls.2018.00570

**Published:** 2018-04-26

**Authors:** Weiwei Wu, Yan Lin, Qianqian Chen, Wenting Peng, Junchu Peng, Jiang Tian, Cuiyue Liang, Hong Liao

**Affiliations:** ^1^Root Biology Center, State Key Laboratory for Conservation and Utilization of Subtropical Agro-bioresources, South China Agricultural University, Guangzhou, China; ^2^Root Biology Center, Fujian Agriculture and Forestry University, Fuzhou, China

**Keywords:** GmSTOP1, proton rhizotoxicity, Al rhizotoxicity, soybean, transcription factor

## Abstract

Proton (H^+^) and aluminum (Al) rhizotoxicity are two major factors limiting crop production in acid soils. Orthologs of the zinc-finger transcription factor, Sensitive To Proton Rhizotoxicity1 (STOP1), have been found to play an essential role in the tolerance to both stresses by regulating the transcription of multiple H^+^ and Al tolerant genes. In the present study, color three *GmSTOP1* homologs were identified in the soybean genome. All three *GmSTOP1* exhibited similar properties as reflected by the harboring of four potential zinc finger domains, localizing in the nucleus, and having transactivation activity. Expression profiling showed that H^+^ stress slightly modulated transcription of all three *GmSTOP1*s, while Al significantly up-regulated *GmSTOP1-1* and *GmSTOP1-3* in root apexes and *GmSTOP1-3* in basal root regions. Furthermore, complementation assays in an Arabidopsis *Atstop1* mutant line overexpressing these *GmSTOP1*s demonstrated that all three GmSTOP1s largely reverse the H^+^ sensitivity of the *Atstop1* mutant and restore the expression of genes involved in H^+^ tolerance. In contrast, only GmSTOP1-1 and GmSTOP1-3 could partially recover Al tolerance in the *Atstop1* mutant. These results suggest that the function of three GmSTOP1s is evolutionarily conserved in H^+^ tolerance, but not in Al tolerance.

## Introduction

Agricultural production is limited on acid soils, which comprise approximately 50% of the world’s potentially arable lands (von Uexküll and Mutert, 1995). There are several constraints limiting plant growth on acid soils, including deficiency of mineral nutrients, such as phosphorus (P), calcium (Ca), and magnesium (Mg), as well as, toxicity of excessive ions, including aluminum (Al^3+^), hydrogen (H^+^), and manganese (Mn^2+^) (Ishitani et al., 2004).

Among these stresses, Al toxicity has been widely acknowledged as a major constraint on crop production (Kochian et al., 2004; Ma, 2007; Bojórquez-Quintal et al., 2017). The Al^3+^ ion can cause rapid and severe impairment of root apical development by damaging cell walls (Horst et al., 1999) and cytoskeletons (Chang et al., 1999; Sivaguru et al., 1999), disturbing DNA and plasma membrane processes (Elstner et al., 1988; Yamaguchi et al., 1999; Meriga et al., 2004), blocking production of callose (Sivaguru et al., 2000), and impeding stress-signaling pathways (Ramos-Díaz et al., 2007). Consequently, plant root growth and nutrient acquisition are inhibited, which leads to significant reductions in crop yields (Ryan et al., 2001; Kochian et al., 2004; Ma, 2007; Bojórquez-Quintal et al., 2017).

Often combined with Al toxicity, H^+^ rhizotoxicity has also been recognized as a major limiting factor for crop production on acid soils (Kochian et al., 2004). When exposed to strong acid conditions, plant root cells will be structurally and functionally damaged (Foy, 1984). For example, obviously swollen root hairs and cracks between cells in root meristems have been observed in Arabidopsis (Koyama et al., 1995, 2001; Iyer-Pascuzzi et al., 2011) and yorkshire-fog grass (*Holcus lanatus*) subjected to acid treatments (Kidd and Proctor, 2001). Moreover, a pH drop from 5.5 to 4.0 is associated with significant membrane depolarization, destruction of epidermal and cortical cells, and, ultimately, inhibition of root growth in *Lotus corniculatus* (Pavlovkin et al., 2009; Palóve-Balang et al., 2012). Similar symptom caused by proton rhizotoxicity have also been observed in many other plant species, such as alfalfa (*Medicago sativa*) (Yokota and Ojima, 1995), spinach (*Spinacia oleracea*) (Yang et al., 2005), common bean (*Phaseolus vulgaris*) (Rangel et al., 2005), and barley (*Hordeum vulgare*) (Song et al., 2011). Besides direct toxicity, low pH can also increase the solubility of other toxic ions, such as Al^3+^, in soil, and thus adversely influence plant root growth. In this aspect, Al and H^+^ toxicities are physiologically linked to one another.

Over the past few decades, mechanisms of plant tolerance to Al and H^+^ rhizotoxicities have been elucidated in many studies. Among them, identification of the C2H2-type zinc finger transcription factor family, STOP1 (Sensitive to Proton Rhizotoxicity1), contributed considerably to understanding of regulatory mechanisms underlying the integration of Al and H^+^ tolerance *in planta* (Iuchi et al., 2007; Yamaji et al., 2009; Ohyama et al., 2013; Sawaki et al., 2014; Fan et al., 2015).

The first *STOP1* gene, *AtSTOP1*, was identified in Arabidopsis (Iuchi et al., 2007). Transcriptome analyses and genetic characterization showed that AtSTOP1 regulates the expression of a set of genes, including three major Al tolerance genes, *AtALMT1* (*Aluminum activated Malate Transporter1*), *AtMATE* (*Multidrug and Toxic Compound Extrusion*), and *AtALS3* (*Aluminum Sensitive3*), along with other genes apparently involved in the regulation of cytosolic pH, such as *GAD1* (*Glutamate Decarboxylase1*), *ME1/2* (*Malic Enzyme1/2*), and *GDH1/2* (*Glutamate Dehydrogenase 1/2*) (Liu et al., 2009; Sawaki et al., 2009; Kobayashi et al., 2014). Interestingly, the STOP1 homolog AtSTOP2, which partially accounts for tolerance to Al and H^+^ rhizotoxicities, is also regulated by AtSTOP1 (Kobayashi et al., 2014). Moreover, a STOP1 ortholog in rice bean (*Vigna umbellata*), *VuSTOP1*, was isolated by suppression subtractive hybridization (Fan et al., 2014). In contrast to the constitutive expression exhibited by *AtSTOP1*, the expression of *VuSTOP1* was inducible by both of Al and H^+^ stresses (Fan et al., 2014). However, the assay of *planta* complementation in *Atstop1* mutant showed that VuSTOP1 could fully restore the transcription of several H^+^-tolerance related genes, but only partially restores the expression of *AtMATE* and *ALS3*, indicating that VuSTOP1 might play a major role in H^+^ tolerance, but only a minor role in Al tolerance (Fan et al., 2015). Similarly, other STOP1 homologs, including NtSTOP1 in tobacco (*Nicotiana tabacum*), LjSTOP1 in *Lotus japonicas*, PnSTOP1 in black poplar (*Populus nigra*), CsSTOP1 in tea (*Camellia sinensis*) and EguSTOP1 in *Eucalyptus* also reportedly possess similar functions in H^+^ tolerance, and only partial or even no functionality in Al tolerance (Ohyama et al., 2013; Sawaki et al., 2014). On the other hand, the mutation of *ART1* (*Al Resistance Transcription Factor1*), a *STOP1* homolog in rice (*Oryza sativa*), appears to only affect Al hypersensitivity (Yamaji et al., 2009). In short, previous studies suggest that STOP1 transcription factors are ubiquitous in plants and have conserved functions in plant stress (Al and/or H^+^) tolerance, though specific responses vary among plant species.

Soybean (*Glycine max*) is one of the most important leguminous crops globally, comprising approximately 68% of crop legume production in the world and 57% of the global oilseed production (Herridge et al., 2008). Though many studies have elucidated the functions of STOP1 orthologs in other plant species, no information is available on whether GmSTOP family members are also involved in H^+^ and Al tolerance in soybean. In the present study, three GmSTOP1 homologs were isolated and characterized from soybean. The function of each *GmSTOP1* gene was analyzed in terms of Al and H^+^ tolerance in Arabidopsis. The results demonstrate that all three *GmSTOP1*s play important roles in H^+^ tolerance, while only GmSTOP1-1 and GmSTOP1-3 could partially recover Al tolerance in Arabidopsis *Atstop1* mutant. Taken together, these results strongly suggest that the three *GmSTOP1*s in soybean share evolutionary conservation of H^+^ tolerance, but not of Al tolerance.

## Materials and Methods

### Plant Material and Growth Conditions

The soybean genotype YC03-3 was chosen as the plant material in this study. Soybean seeds were germinated in paper rolls moistened with modified one-half-strength nutrient solution as previously described (Liang et al., 2013). The resultant seedlings were then gown in full strength nutrient solution for 24 h before being used for various treatments. For the low pH treatment, soybean seedlings were subjected to 0.5 mM CaCl_2_ (pH 4.2) for 0, 2, 4, 6, and 12 h. After low pH treatment, root tips (0–2 cm) were harvested for gene expression assays. For the tissue specific expression experiment, soybean root tips (0–2 cm), which was further divided into two segments (0–1 cm and 1–2 cm), basal roots (>2 cm) and leaves were harvested after 4 h of Al (0 or 50 μM AlCl_3_ in 0.5 mM CaCl_2_, pH 4.2) treatment. For the Al dose experiment, soybean seedlings were treated with 0, 10, 50, and 100 μM AlCl_3_ in 0.5 mM CaCl_2_ solution (pH 4.2) for 4 h. For the time-course experiment, soybean seedlings were transplanted to Al (50 μM AlCl_3_ in 0.5 mM CaCl_2_, pH 4.2) treatments for 0, 2, 4, 6, and 12 h. In both of the concentration response experiment and time-course experiment, root tips (0–2 cm) were separately harvested for gene expression assays. All experiments had four biological replicates.

### Phylogenetic Analysis and Characterization of GmSTOP1 Proteins in Soybean

TBLASTN analysis using the AtSTOP1 proteins sequence (accession number: Q9C8N5.1) as the query sequences was conducted at the Phytozome website^1^. Consequently, three STOP1 homologs with high similarity to AtSTOP1 were identified and designated as GmSTOP1-1 (Glyma10g35940), GmSTOP1-2 (Glyma16g27280), and GmSTOP1-3 (Glyma20g31650). Subsequently, multiple sequence alignment and phylogenetic tree construction were conducted using the deduced protein sequences of all three GmSTOP1s together with other STOP1 homologs, including AtSTOP1 from Arabidopsis, NtSTOP1 from tobacco, LjSTOP1 from *Lotus japonicas*, PnSTOP1 from black poplar (*Populus nigra*), CsSTOP1 from tea, EguSTOP1 from *Eucalyptus*, OsART1 from rice, PpSTOP1 from *Physcomitrella patens*, and TaSTOP1-A, TaSTOP1-B, TaSTOP1-D from wheat (*Triticum aestivum*). ClustalX2 and MEGA4.1 were used for the multiple sequence alignment and phylogenetic tree construction, respectively. The phylogenetic tree was constructed using the Neighbor-Joining method with 1,000 bootstrap replicates.

### RNA Extraction and Quantitative Real-Time PCR

Total RNA was extracted from plant tissues using RNA-solve reagent (OMEGA Bio-Tek, Norcross, GA, United States). Genomic DNA in the RNA samples was eliminated with RNase-free DNase I (Invitrogen, Carlsbad, CA, United States). The resulting extracts were then used to conduct the reverse transcription via MMLV-reverse transcriptase (Promega, Madison, WI, United States) following the manufacturer’s instructions. Subsequently, SYBR Green monitored qRT-PCR (quantitative real-time PCR) analysis was performed using a ABI Step-one Plus real-time PCR system (Thermo Fisher Scientific, Waltham, MA, United States). The primer pairs used for expression analysis are listed in Supplementary Table S1.

### Subcellular Localization of GmSTOP1s

The full length cDNAs of the three identified *GmSTOP1* genes were amplified from first strand cDNA derived from soybean roots using gene specific primer pairs as listed in Supplementary Table S1. The PCR products were then cloned into the pMD18-T vector (Takara, Japan) for sequence confirmation.

Full-length cDNA of the three *GmSTOP1*s was fused with enhanced green fluorescent protein (GFP) to construct *35S::GmSTOP1*s*-GFP* plasmids. Each construct was introduced into tobacco (*Nicotiana tabacum*) leaf cells according to previously described methods (Liang et al., 2010; Liu et al., 2016). The *35S::GFP* construct was used as the control. Fluorescence signals of GFP were detected at 488 nm by confocal scanning microscope (LSM780; Zeiss, Germany).

### Transcriptional Activation Activity of GmSTOP1s

Gene specific primers with terminal *Sfi*I and *Sal*I restriction sites were used to amplify full-length cDNA of GmSTOP1-1 and GmSTOP1-3, whereas, primers with *BamH*I and *Pst*I restriction sites were used to amplify full-length cDNA of GmSTOP1-2. Sequence fragments were digested by the corresponding restriction enzymes and inserted into the pGBKT7 vector (Clontech, Japan), producing *pGBKT7-GmSTOP1*s plasmids. The resultant plasmids and the *pGBKT7* empty vector were then transformed into yeast strain AH109. After verification by PCR, transformed AH109 cells were cultured on either SD-Trp or SD-His medium for 3 days. The yeast cells grown on SD-Trp were then printed onto filter paper moistened with X-gal solution. Subsequently, the filter paper was freeze-thawed with liquid nitrogen and moistened again with X-gal solution. The appearance of blue areas on the filter paper was used to determine β-galactosidase activity.

### Complementation of GmSTOP1s in *Atstop1* Mutant Plants

The three identified *GmSTOP1*s were separately introduced into the modified *pBEGFP* binary vector under the control of a *35S CaMV* promoter to produce an over-expression construct that was then transformed into *A. tumefaciens* strain Gv3101. Subsequently, the constructs were transformed into Arabidopsis *Atstop1* mutant plants via the floral dip method (Clough and Bent, 1998). Two independent over-expression lines for each gene were verified by qRT-PCR and used for further analysis as complemented lines.

To investigate the functions of *GmSTOP1*s in resistance to H^+^ and Al toxicity, wild type, *Atstop1* mutant and the complemented lines overexpressing *GmSTOP1* were germinated on solid Murashige and Skoog (MS) medium for 5 days. Uniform seedlings with ∼1.5 cm root lengths were transferred to modified 1/30 strength Hoagland nutrient solution (without NH_4_H_2_PO_4_ and plus 1 mM CaCl_2_) with different treatments as described (Fan et al., 2015). Control plants were grown in media with pH adjusted to 5.8, while treated plants were grown in low pH media (pH 4.7) or media containing 2 μM AlCl_3_ (pH 5.0) for 7 days (Fan et al., 2015). Upon harvest, roots of each plant were scanned and analyzed in Image J (National Institutes of Health, United States). All experiments had four biological replicates, each of which contains two plants.

For analysis of H^+^ and Al genes expression responses, uniform Arabidopsis seedlings were treated with 1/30 strength Hoagland nutrient solution containing 2 μM AlCl_3_ (pH 5.0) and low pH (pH 4.7) for 24 h (Fan et al., 2015). All experiments were conducted in a growth incubator running a 24°C, 12h/22°C, 12 h day/night cycle. The whole roots were harvested for gene expression assays. The primer pairs of target genes for qRT-PCR analysis are listed in Supplementary Table S1. Arabidopsis *UBQ1* was used as housekeeping gene control to normalize the expression of the corresponding genes. All experiments had four biological replicates.

### Statistical Analysis

All data were analyzed by Student’s *t*-tests using SPSS 13.0 (SPSS Institute, Chicago, IL, United States).

## Results

### Identification of STOP1 Homologs in Soybean

**FIGURE 1:**
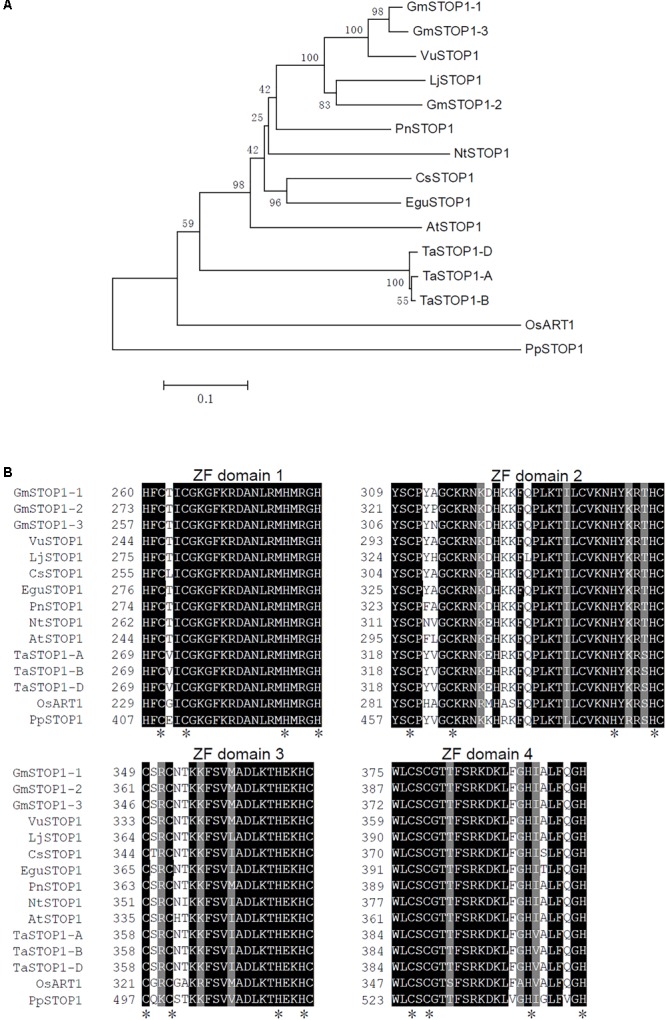
Phylogenetic tree and amino acid alignments of predicted C_2_H_2_ zinc finger domains in plant STOP1s. **(A)** Phylogenetic tree was generated based on an amino-acid alignment with STOP1 orthologs from several plant species. **(B)** Alignment of the amino acid sequences of predicted C2H2 zinc finger domains in STOP1 proteins. Black background indicates identical residues. Asterisks indicate conserved Cys and His residues of C2H2 motifs. The plant STOP1 proteins aligned include representatives from *Glycine max* (GmSTOP1-1, XP_006588359.1; GmSTOP1-2, XP_006598713.1; GmSTOP1-3, XP_014628358.1), *Arabidopsis thaliana* (AtSTOP1, NP_174697.1), *Nicotiana tabacum* (NtSTOP1, AB811781), *Lotus japonicus* (LjSTOP1, BAN67817.1), *Vigna umbellata* (VuSTOP1, KP637172), *Camellia sinensis* (CsSTOP1, BAN67815.1), *Populus nigra* (PnSTOP1, BAN67813.1), *Eucalyptu*s (EguSTOP1, BAO56822.1), *Triticum aestivum* (TaSTOP1-A, AGS15201.1; TaSTOP1-B, AGS15202.1; TaSTOP1-D, AGS15195.1), *Oryza sativa* (OsART1, AB379846), and *Physcomitrella patens* (PpSTOP1, BAN67814.1).

A homolog search resulted in retrieval of three STOP1 homologs in the soybean genome, which were named GmSTOP1-1 (Glyma.10G215200), GmSTOP1-2 (Glyma.16G156400) and GmSTOP1-3 (Glyma.20G176500) based on genome localization. A phylogenetic tree showed that the STOP1 homologs in dicots were differentiated from those in monocots (**Figure 1A**). Moreover, GmSTOP1-1 and GmSTOP1-3 present as duplicated pair and display high similarity with VuSTOP from rice bean, while GmSTOP1-2 clusters in another sub-clade with LjSTOP1 from *Lotus japonicus* (**Figure 1A**). Moreover, the deduced amino acid sequences of all three GmSTOP1s contain four putative C2H2 zinc finger domains that are highly conserved in STOP1 orthologs from other plant species (**Figure 1B**).

### Subcellular Localization and Transcription Activation Activity of GmSTOP1s

**FIGURE 2:**
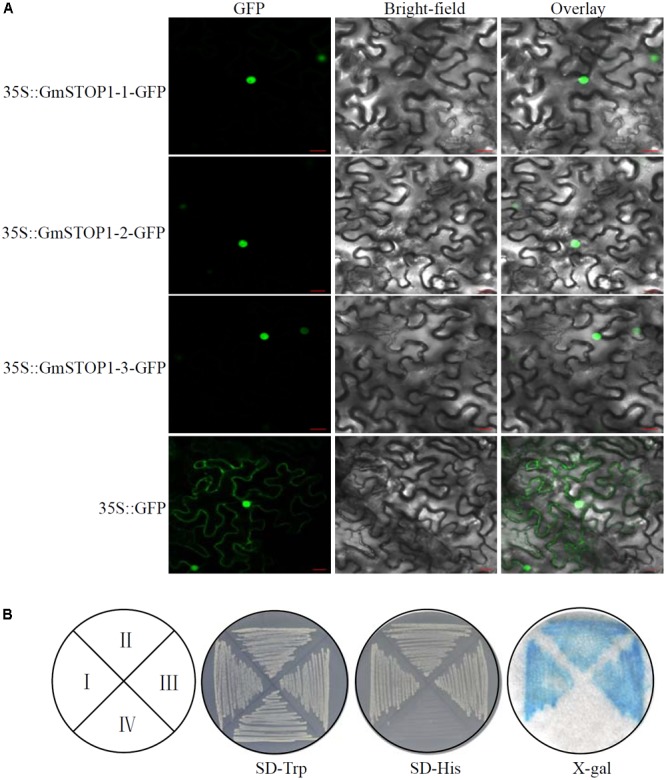
Subcellular localization and transactivation assay of GmSTOP1s. **(A)** *35S::GmSTOP1*s*-GFP* and *35S::GFP* (control) constructs were introduced individually into *Nicotiana benthamiana* leaves. Scale bars: 20 μm. **(B)** Transactivation assay of GmSTOP1-1 (I), GmSTOP1-2 (II), GmSTOP1-3 (III), and control (IV). β-galactosidase activity is indicated by blue color on the filter paper using X-gal as the substrate.

To determine the subcellular localization of the three identified GmSTOP1s, *GmSTOP1*-*GFP* fusion constructs were assayed for transient expression in tobacco leaf cells. The results showed that control GFP fluorescence was detectable in both the nucleus and cytoplasm. In contrast, fluorescence derived from GmSTOP1-GFP constructs was exclusively localized within the nucleus (**Figure 2A**), strongly suggesting that the three GmSTOP1 members are all nucleus localized proteins.

Transcription activity of the three GmSTOP1 members was determined in a one-hybridization expression system in yeast. The results showed that the yeast strain AH109 transformed with either a *pGBKT7-GmSTOP1* or the *pGBKT7* empty vector could grow well on the SD-Trp medium (**Figure 2B**). However, only the three AH109 strains transformed with *pGBKT7-GmSTOP1*s grew well on the SD-His medium (**Figure 2B**). Furthermore, all three of the AH109 strains transformed with a *pGBKT7-GmSTOP1* showed high β-galactosidase activity as indicated by the blue color on filter paper using X-gal as a substrate (**Figure 2B**). Therefore, the ability of all three GmSTOP1 homologs to activate *lacZ* expression strongly suggests that each one functions as a transcription factor.

### Expression Patterns of *GmSTOP1*s in Response to Al and Low pH Stress

**FIGURE 3:**
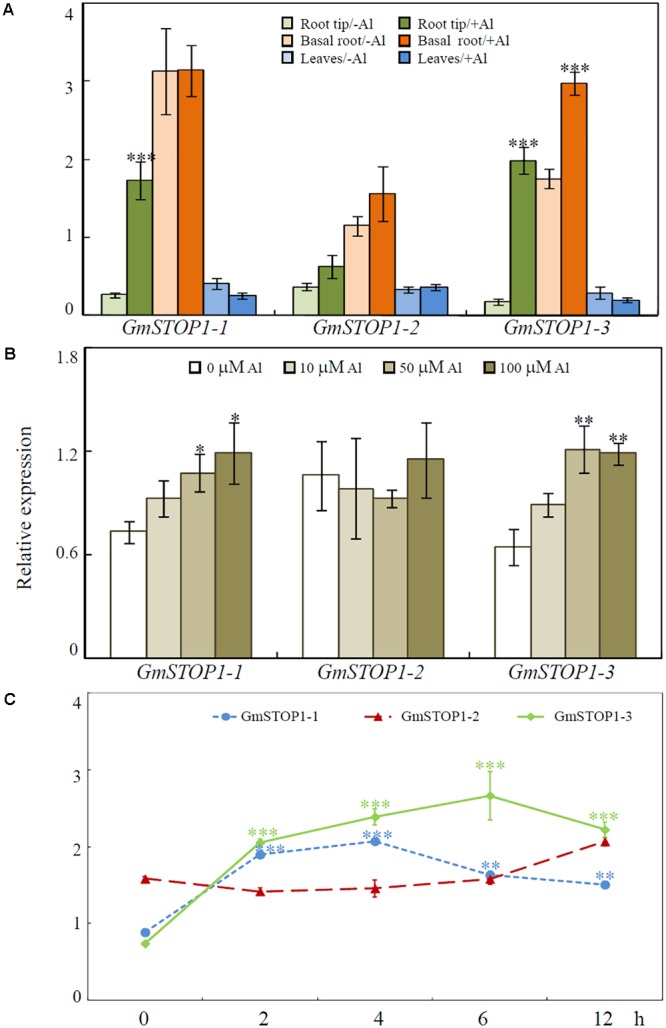
Expression patterns of *GmSTOP1*s in response to Al toxicity. **(A)** Relative expression of *GmSTOP1*s in root tips (0–2 cm), basal roots (>2 cm) and leaves after 4 h of –Al (0 μM) or +Al (50 μM) treatment. **(B)** Relative expression of *GmSTOP1*s in soybean root tips (0–2 cm) treated with different concentrations of Al for 4 h. **(C)** Relative expression of *GmSTOP1*s in soybean roots tips (0–2 cm) in response to Al (50 μM) for different treatment times. Asterisks indicate significant differences between the +Al treatment and –Al control (^∗^0.01 < *P* < 0.05; ^∗∗^0.001 < *P* < 0.01; ^∗∗∗^*P* < 0.001).

Quantitative real-time PCR was used to analyze *GmSTOP1* expression patterns in soybean seedlings. Expression levels of the three *GmSTOP1*s were hardly affected by low pH stress during the 12 h treatment period (Supplementary Figure S2). On the other hand, transcriptional responses varied among the three *GmSTOP1* genes in response to Al stress. As shown in **Figure 3A**, transcription of *GmSTOP1-2* was not significantly affected after 4 h of Al treatment in any tissues, including root tips (0–2 cm), basal regions of roots (>2 cm) and leaves, (**Figure 3A**). In contrast to the constitutive expression of *GmSTOP1-2* in roots, transcript levels of *GmSTOP1-1* and *GmSTOP1-3* increased by more than 6- and 11-fold, respectively, in root tips after 4 h of Al treatment (**Figure 3A**). However, in root basal regions, only transcription of *GmSTOP1-3* increased by more than 1.7-fold in response to Al stress, while no detectable change was observed for *GmSTOP1-1*.

Dose-responses of *GmSTOP1*s to Al stress were further analyzed in soybean root tips after 4 h of Al treatment. Transcript accumulations of both *GmSTOP1-1* and *GmSTOP1-3* were strictly dependent on Al concentration in the medium (**Figure 3B**), with transcript abundances enhanced for both *GmSTOP1-1* and *GmSTOP1-3* in 50 and 100 μM Al treatments (**Figure 3B**). The expression of *GmSTOP1-2* was constitutively expressed at relatively high levels regardless the external Al concentrations (**Figure 3B**).

Results from time-course experiments showed that the expression of both *GmSTOP1-1* and *GmSTOP1-3* were quickly enhanced in response to Al stress by more than twofold after 2 h of Al treatment, and remained high over 12 h (**Figure 3C**). Meanwhile, the expression of *GmSTOP1-2* did not vary during the period of Al treatment (**Figure 3C**).

### Functional Analysis of GmSTOP1s in the Arabidopsis *Atstop1* Mutant

**FIGURE 4:**
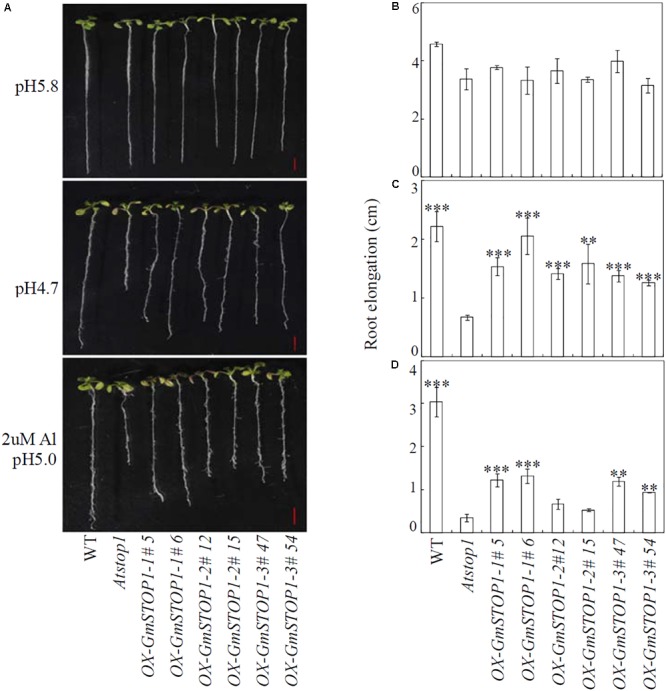
Effects of low pH and Al stresses on the growth of Arabidopsis *Atstop1* mutant plants overexpressing *GmSTOP1*s. Uniform seedlings with ∼1.5 cm root lengths were treated with low pH (pH 4.7) and Al stresses (2 μM Al and pH 5.0) for 7 days. The phenotypes of wild-type (WT), *Atstop1* and *GmSTOP1* overexpressing *Atstop1* lines in response to H^+^ and Al were photographed **(A)**. Root elongation for each line was measured under control **(B)**, low pH **(C)**, and Al **(D)** treatments. Each bar represents the mean of four biological replicates with standard error. Asterisks indicate significant differences in comparison to the *Atstop1* mutant (^∗^0.01 < *P* < 0.05; ^∗∗^0.001 < *P* < 0.01; ^∗∗∗^*P* < 0.001). Scale bars: 5 mm.

In order to examine their functions in plant H^+^ and Al tolerance, all three *GmSTOP1*s were overexpressed in the Arabidopsis *Atstop1* mutant. The expression of all three of the *GmSTOP1* genes in the *Atstop1* mutant was verified by qRT-PCR. Under normal growth conditions, no significant differences were observed among wild type, *Atstop1* mutant and complemented lines overexpressing any of the *GmSTOP1*s (**Figures 4A,B**). However, under low pH condition (pH 4.7), root elongation of wild type and *Atstop1* mutant was inhibited by 51% and 80%, respectively (**Figures 4A,C**). In each of two complemented lines of *GmSTOP1-1* (#5 and #6), *GmSTOP1-2* (#12 and #15) and *GmSTOP1-3* (#47 and #54), root elongation was inhibited much less than that of the *Atstop1* mutant (**Figures 4A,C**). These results suggest that all three *GmSTOP1*s are able to confer H^+^ tolerance in *Atstop1* mutant plants.

Addition of Al to the low pH culture solution slightly decreased root elongation of wild type plants, but significantly inhibited root elongation of *Atstop1* mutants as indicated by a 90% decrease in root elongation compared to root elongation in wild type plants (**Figures 4A,D**). Unlike the role of *GmSTOP1*s in H^+^ tolerance, the functions of *GmSTOP1*s in Al tolerance varied. Each of the lines complemented with *GmSTOP1-1* (#5 and #6) and *GmSTOP1-3* (#47 and #54) overexpression recovered elongation to 28% and 32%, and 29% and 19% of that of wild type, respectively (**Figures 4A,D**). In contrast, lines complemented with *GmSTOP1-2* overexpression did not recover root elongation. These results indicate that *GmSTOP1-1* and *GmSTOP1-3*, but not *GmSTOP1-2* can partially reverse the Al hypersensitivity of *Atstop1* mutant plants.

### Transcription of STOP1 Down-Stream Genes in *GmSTOP1* Complemented *Atstop1* Mutants

**FIGURE 5:**
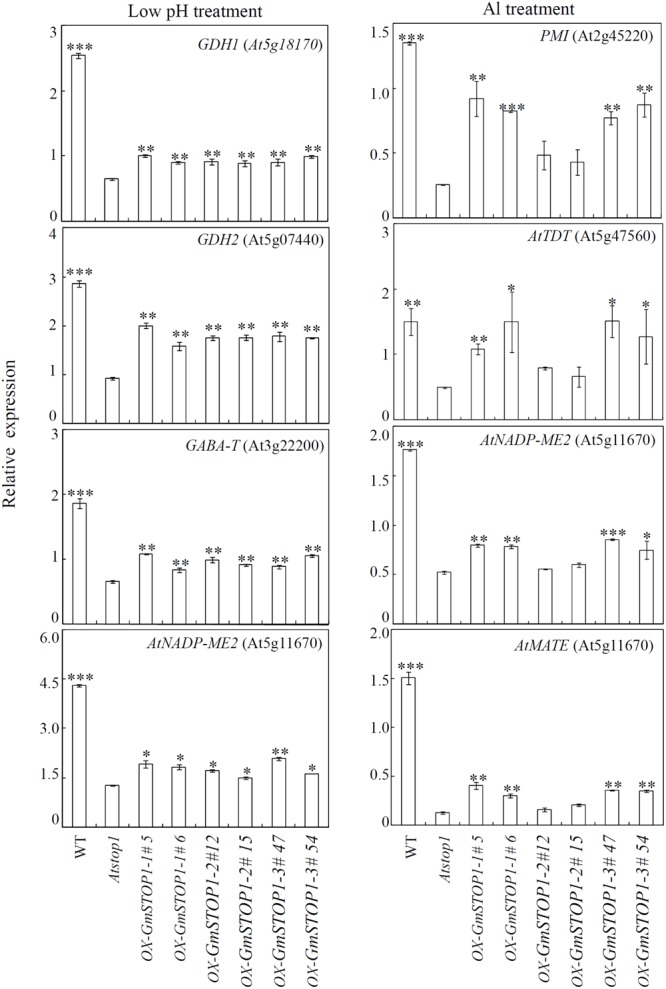
Transcriptional accumulation of genes regulated by *GmSTOP1*s under low pH and Al stresses. Wild-type (WT), *Atstop1* mutant and complemented lines overexpressing *GmSTOP1-1* (#5 and #6), *GmSTOP1-2* (#12 and #15), and *GmSTOP1-3* (#47 and #54) were exposed to low pH (pH 4.7) and Al treatments (AlCl_3_: 2 μM; pH 5.0) for 24 h. Transcript abundances of *GDH1, GDH2, GABA-T* and *AtNADP-ME2* were quantified from plants grown in the low pH treatment, while expression levels of *PMI* (At2g45220), *AtTDT* (At5g47560), *AtNADP-ME2* (At5g11670), and *AtMATE* (At1g51340) were quantified in Al treated samples. UBQ1 transcript levels were used as the internal standard. Data are expressed as means of four replicates. Asterisks indicate significant differences in comparison to the *Atstop1* mutant (^∗^0.01 < *P* < 0.05; ^∗∗^0.001 < *P* < 0.01; ^∗∗∗^*P* < 0.001).

The differential contributions of *GmSTOP1*s to H^+^ and Al tolerance were further determined by investigating the transcript levels of several related down-stream genes in *GmSTOP1* complemented *Atstop1* mutants. The results showed that expression of several H^+^ tolerance genes were significantly restored in all of the complemented *Atstop1* mutant lines (**Figure 5**). Among responsive genes, the expression of *GDH2* (At5g07440) was restored the most, with transcription returning to at least 50% of transcript levels in WT plants (**Figure 5**). Although restored to lesser extents, the expression of three other H^+^ tolerance genes, *GDH1* (At5g18170), *GABA-T* (At3g22200) and *NADP-malate enzym*e 2 (*NADP-ME2*), was restored nonetheless by 37, 25, and 20%, respectively, over expression in the *Atstop1* mutant (**Figure 5**).

Unlike expression patterns of H^+^ tolerance related genes, expression responses of Al tolerance related genes varied among *GmSTOP1* complemented lines. These Al tolerance related genes included *pectin methylesterase inhibitor superfamily protein* (*PMI, At2g45220*), *AtTDT* (At5g47560), *NADP-ME2* (At5g11670), and *AtMATE* (At1g51340). In both *GmSTOP1-1* and *GmSTOP1-3* complemented lines, the expression of *AtPMI, AtTDT, NADP-ME2*, and *AtMATE* were partially restored. Yet, in *GmSTOP1-2* complemented lines, the expression of each of these four genes was not affected relative to expression in *Atstop1* mutants (**Figure 5**).

## Discussion

Proton and Al rhizotoxicities are two of the major constraints of plant growth and development on acid soil (Kochian et al., 2004). Plants have adapted to these stresses by developing a variety of coping strategies involving a number of genes (Liu et al., 2014; Kochian et al., 2015). Recent studies have revealed that H^+^ and Al tolerance mechanisms are regulated by STOP1 transcription factors in many plant species (Iuchi et al., 2007; Sawaki et al., 2009, 2014; Yamaji et al., 2009; Garcia-Oliveira et al., 2013; Ohyama et al., 2013; Fan et al., 2015). However, few studies have attempted to systematically dissect the possible roles of all STOP1 members in a single species responding to H^+^ stress, Al toxicity, or both.

In the present study, a total of three *GmSTOP1* genes were identified in the soybean genome. All of these GmSTOP1 homologs are localized to the nucleus, and exhibit transcription activity (**Figure 2**). Sequence analysis revealed that GmSTOP1-2 is phylogenetically distinct from GmSTOP1-1 and GmSTOP1-3, which appear to be a duplicated pair (**Figure 1**). As gene duplication provides opportunities for functional divergence (Force et al., 1999; Lynch et al., 2001; Flagel and Wendel, 2009; Libault et al., 2010; Schmutz et al., 2010), we hypothesize that the three *GmSTOP1* genes might have divergent functions in regard to H^+^ and Al tolerance even though they are highly conserved in some features (**Figures 1, 2**).

To dissect the differential contributions of *GmSTOP1*s in H^+^ tolerance, expression analysis was conducted. The results showed that similar to *AtSTOP1* in Arabidopsis (Iuchi et al., 2007), all three *GmSTOP1* genes were constitutively expressed and hardly affected by H^+^ treatment (Supplementary Figure S2). Further complementation assays showed that all three *GmSTOP1* genes are able to confer H^+^ tolerance to the Arabidopsis *Atstop1* mutant (**Figure 4C**). These results are consistent with the previous studies reporting that *STOP1* orthologs in dicots are able to confer H^+^ tolerance to the H^+^ sensitive *Atstop1* mutant (Ohyama et al., 2013; Sawaki et al., 2014; Fan et al., 2015). Furthermore, the H^+^ hypersensitivity of the *Atstop1* mutant is the result of down-regulation of genes in several pH regulation pathways caused by the dysfunction of AtSTOP1 (Sawaki et al., 2009).

The expression of several H^+^ tolerance genes, including *STOP2, CIPK23*, and *PGIP1*, has been restored *in planta* in complementation assays of *Atstop1* by *STOP1* orthologs from rice bean (*VuSTOP1*), *Eucalyptus* (*EguSTOP1*), tobacco (*NtSTOP1*), black poplar (*PnSTOP1*), tea (*CsSTOP1*), *Lotus japonicus* (*LjSTOP1*), or *Physcomitrella patens* (PpSTOP1) (Ohyama et al., 2013; Sawaki et al., 2014; Fan et al., 2015). However, none of these genes were affected by complementation with any of the *GmSTOP1* homologs in the *Atstop1* mutant (Supplementary Figure S4). Instead, *GmSTOP1* complementation restored the transcription of several other H^+^ tolerance relative genes, including *GDH1, GDH2, GABA-T*, and *AtNADP-ME2*, which are considered to play roles in maintaining pH homeostasis in plants. For example, *AtNADP-ME2* has been reported to function in the pH stat pathway through consumption of cytosolic H^+^ (Roberts et al., 1992; Sakano, 1998). Meanwhile, *GDH1, GDH2* and *GABA-T* are the major isoforms in the “GABA shunt” pathway, which contributes largely to cytosolic pH homeostasis in plant cells (Crawford et al., 1994; Bown and Shelp, 1997). These results strongly suggest that all three *GmSTOP1* homologs participate in conserved functions in H^+^ tolerance mainly through regulation of similar pH stat pathways that are distinct from the pH stat pathways regulated by other plant *STOP1* orthologs (Ohyama et al., 2013; Sawaki et al., 2014; Fan et al., 2015).

Interestingly, in the presence of Al, expression of both *GmSTOP1-1* and *GmSTOP1-3* quickly escalated in root tips, while expression of *GmSTOP1-2* was not significantly affected (**Figure 3** and Supplementary Figure S3). Similar results have also been reported for bread wheat, in which expression of *TaSTOP1-A* was found to be responsive to H^+^ and Al stresses and divergent from the responses of *TaSTOP1-B* and *TaSTOP1-D* (Garcia-Oliveira et al., 2013). It has been suggested that this divergence might be mainly due to the presence of a pyrimidine-rich stretch and the absence of a light responsive element in the 5′ UTR of TaSTOP1-A compared to its homologs TaSTOP1-B and TaSTOP1-D (Garcia-Oliveira et al., 2013). Consistent with this, our investigation revealed that the 5′-UTR is more similar between *GmSTOP1-1* and *GmSTOP1-3* than it is between either of these genes and *GmSTOP1-2* (Supplementary Figure S1). Therefore, there is a possibility that the differential expression between *GmSTOP1-2* and the other two *GmSTOP1*s in response to Al stress might be due to divergence in the 5′-UTR. Thereby, divergence between *GmSTOP1-2* and the other two *GmSTOP1*s in both transcriptional regulation and protein sequence further suggests that *GmSTOP1-2* functions differently than *GmSTOP1-1* and *GmSTOP1-3* in Al tolerance responses.

Even more evidence in support of divergence among *GmSTOP1*s in Al tolerance functionality was gathered in complementation assays (**Figure 4**). The results strongly indicate that *GmSTOP1-1* and *GmSTOP1-3* are involved at least partially in *AtSTOP1* related Al tolerance responses, whereas GmSTOP1-2 is not. It has been reported that AtSTOP1 regulates transcription of three major Al tolerance genes in Arabidopsis, namely *AtALMT1, ALS3*, and *AtMATE* (Kobayashi et al., 2007; Liu et al., 2009; Sawaki et al., 2009; Tokizawa et al., 2015). Among them, *AtALMT1* accounts for more than 70% of the Al tolerance phenotype in Arabidopsis (Iuchi et al., 2007; Liu et al., 2012). In the current study, it was interesting to find that none of the *GmSTOP1* homologs restores the expression of *AtALMT1* or *ALS3* in the *Atstop1* mutant, while *AtMATE* expression was recovered slightly in both *GmSTOP1-1* and *GmSTOP1-3* complemented lines, but not in *GmSTOP1-2* complemented lines (**Figure 5** and Supplementary Figure S4). Similar results were also reported in other plant species, where most STOP1 orthologs are not able to restore the expression of all three Al tolerance genes in the *Atstop1* mutant. For example, LjSTOP1, CsSTOP1, and PnSTOP1 can slightly restore the expression of *AtALMT1*, but not the expression of *ALS3* or *AtMATE*, while VuSTOP1 can partially restore the expression of *ALS3* and *AtMATE*, but not the expression of *AtALMT1* (Ohyama et al., 2013; Fan et al., 2015). Placing the current results in the context of previous reports suggests that the regulatory functions of *AtSTOP1* in Al tolerance is not entirely conserved among plant *STOP1* orthologs.

Potential phenotypic effects of GmSTOP1-1/GmSTOP1-3 in the *Atstop1* mutant in response to Al toxicity are revealed by considering functions of *PMI, AtTDT*, and *NADP-ME2*, which are down-regulated in *Atstop1* mutants subjected to Al stress (Sawaki et al., 2009), and which had expression restored in the complementation experiments herein. Members of the PMI family have been reported to inhibit pectin methylesterase activity, and thereby increasing Al tolerance (Sénéchal et al., 2015; Geng et al., 2017). The other two genes, *AtTDT* and *NADP-ME2*, are involved in malate homeostasis and metabolism in the vacuole and cytosol, respectively (Hurth et al., 2005; Badia et al., 2015). Results in the present study showed that all of these genes were partially restored in both *GmSTOP1-1* and *GmSTOP1-3* complemented lines, but not in *GmSTOP1-2* complemented lines (**Figure 5**). These expression responses are in accord with the variation in Al tolerance observed among *GmSTOP1*s complemented lines (**Figure 4D**). Therefore, it appears that GmSTOP1-1/GmSTOP1-3 might function in Al tolerance through the regulation of cell wall modifications and malate metabolism.

Overall, the present study identifies three *GmSTOP1* homologs in the soybean genome, all of which localize in the nucleus and have the transactivation potential. Complementation assays suggest that all three *GmSTOP1* homologs play major roles in H^+^ tolerance through transcriptional regulation of H^+^ tolerance genes, whereas, only GmSTOP1-1 and GmSTOP1-3 function in Al tolerance. Taken together, the results herein suggest that the functions of the three identified *GmSTOP1*s are evolutionarily conserved in H^+^ tolerance responses, but not in Al tolerance responses.

## Author Contributions

WW, YL, QC, WP, JP, and CL performed the experiments and collected the data. CL, JT, and HL designed the research, analyzed the data, and wrote the manuscript.

## Conflict of Interest Statement

The authors declare that the research was conducted in the absence of any commercial or financial relationships that could be construed as a potential conflict of interest.

## Footnotes

1 https://phytozome.jgi.doe.gov/pz/portal.html
