# Supplementary material for: Functional Conservation and Divergence of Soybean GmSTOP1 Members in Proton and Aluminum Tolerance
Source: Front Plant Sci. 2018 Apr 26;9:570. doi: 10.3389/fpls.2018.00570 (PMC5932199; doi:10.3389/fpls.2018.00570)
Supplement: Supplementary file 1 [file Table_1.PDF]

Table S1. Primer pairs used in the study

Purpose	*Gene*	Forward primers (5'-3')	Backward primers (5'-3')
GmSTOP1s over-expression	*GmSTOP1-1*	CTGATCTAGATCTCTGCGTTTAAAATGGATCC	CTGAGGTACCCATTATTATAAATTGTCGCTGTC
	GmSTOP1-2	GTCAGTCGACATGGATTCAAATGGGAGCCTAC	GACTGGATCCTTATAAAAGATTGTCAGAACTAGATTCTCC
	GmSTOP1-3	CTGATCTAGAATGGATCCAAAAGGAAGCTTATGTGC	ACATGAATTCTTACTGGTTGGCAAATT
GmSTOP1s subcellular localization	*GmSTOP1-1*	CTGATCTAGATCTCTGCGTTTAAAATGGATCC	CTGAGGTACCTTGTCGCTGTCAAATTTGGG
	GmSTOP1-2	GTCAGTCGACATGGATTCAAATGGGAGCCTAC	GACTGGATCCAGATTGTCAGAACTAGATTCTCC
	GmSTOP1-3	CTGATCTAGAATGGATCCAAAAGGAAGCTTATGTGC	CTGAGGTACCAGATTGTCAGAACTTGATTCACCG
GmSTOP1s transcriptional activation	*GmSTOP1-1*	GACTGGCCATGGAGGCCATGGATCCAAAAGGAAGCTTATGTGC	GTACGTCGACTTATAAATTGTCGCTGTCAAATTTGG
	GmSTOP1-2	GTACGGATCCAAATGGATTCAAATGGGAGCCTAC	CAGTCTGCAGTTATAAAAGATTGTCAGAACTAGATTCTCC
	GmSTOP1-3	GACTGGCCATGGAGGCCATGGATCCAAAAGGAAGCTTATGTGC	GTACGTCGACCTACAGATTGTCAGAACTTGATTCACC
Expression pattern of *GmSTOP1s*	*GmSTOP1-1*	AAGTGGAGCATTCAGGTTCCGTTGG	GGAAGAGGTCCTTGTGCAGTTTTTAGC
	GmSTOP1-2	TAGCTGAGGCACCCATTCATGATAG	TCTGGGTCATCAAATAGAGGTCTGG
	GmSTOP1-3	GCATTCAGGTTGTTAGAGCGTCC	GGTTAAGGAAGAGGACCTTGTAGCAC
	GmEF-1α	TGCAAAGGAGGCTGCTAACT	CAGCATCACCGTTCTTCAAA
Expression parttern of H+-tolerance and Al-tolerance gene in Arabidopsis	*AtGDH1*	GAAGGTGAACGATGAGCTAAAGACT	GCGTCTTCTAGTACTTGTAAACACA
	GABA-T	TGTTAGTCCGTGTTGCAGGTGA	CACTTCTTGTGCTGAGCCTTGA
	GDH2	AGGAGTGACAGTGAGTTACTTCG	TGGCTCGAGCGACTCGGTTA
	NADP-ME2	GCACAAGTGACAGAAGAGCATTAC	GAGAGTTCACAAACAGCATCATAATG
	AtCIPK23	CAGTCGACATAAGCTGTTTACTTTT	GAATCTCAGCGTCCGACAATATAGAA
	AtPGIP1	TAAACCAAGCTTATCTCTAGGATTA	CCATCAAATAAAACATTTTGAAAATGTGC
	AtSTOP2	CGTTTCTTTGTTTTTGGGCCACGTA	TCTATGCATGATACCCATCAGAACC
	PMI	ACTAGGACCAAAGTGCCGTG	TTTGCGCGTCGAGTTTTGAG
	AtTDT	GCATATAGAGATCAAAGACATGATC	GACCTCATCTAAATTTCCCATTCCA
	AtALMT1	GGCCGACCGTGCTATACGAG	CATGAGTCCTGTGAACTCCC
	AtMATE	ACCATGAGTCGATGAGAGGAAGAG	TATCGATCCTTGCCGGGACTTCA
	ALS3	TATCGATCCTTGCCGGGACTTCA	GCTTGTCTTGGCGTTGCTCCTA
	UBQ1	AGAGCTGTCAACTGCAGGAAGAA	ACAAGAAAAACAAACCCTATCAAAGG
