# Supplementary material for: Functional Conservation and Divergence of Soybean GmSTOP1 Members in Proton and Aluminum Tolerance
Source: Front Plant Sci. 2018 Apr 26;9:570. doi: 10.3389/fpls.2018.00570 (PMC5932199; doi:10.3389/fpls.2018.00570)
Supplement: Supplementary file 2 [file Image_1.PDF]

Fig S1.

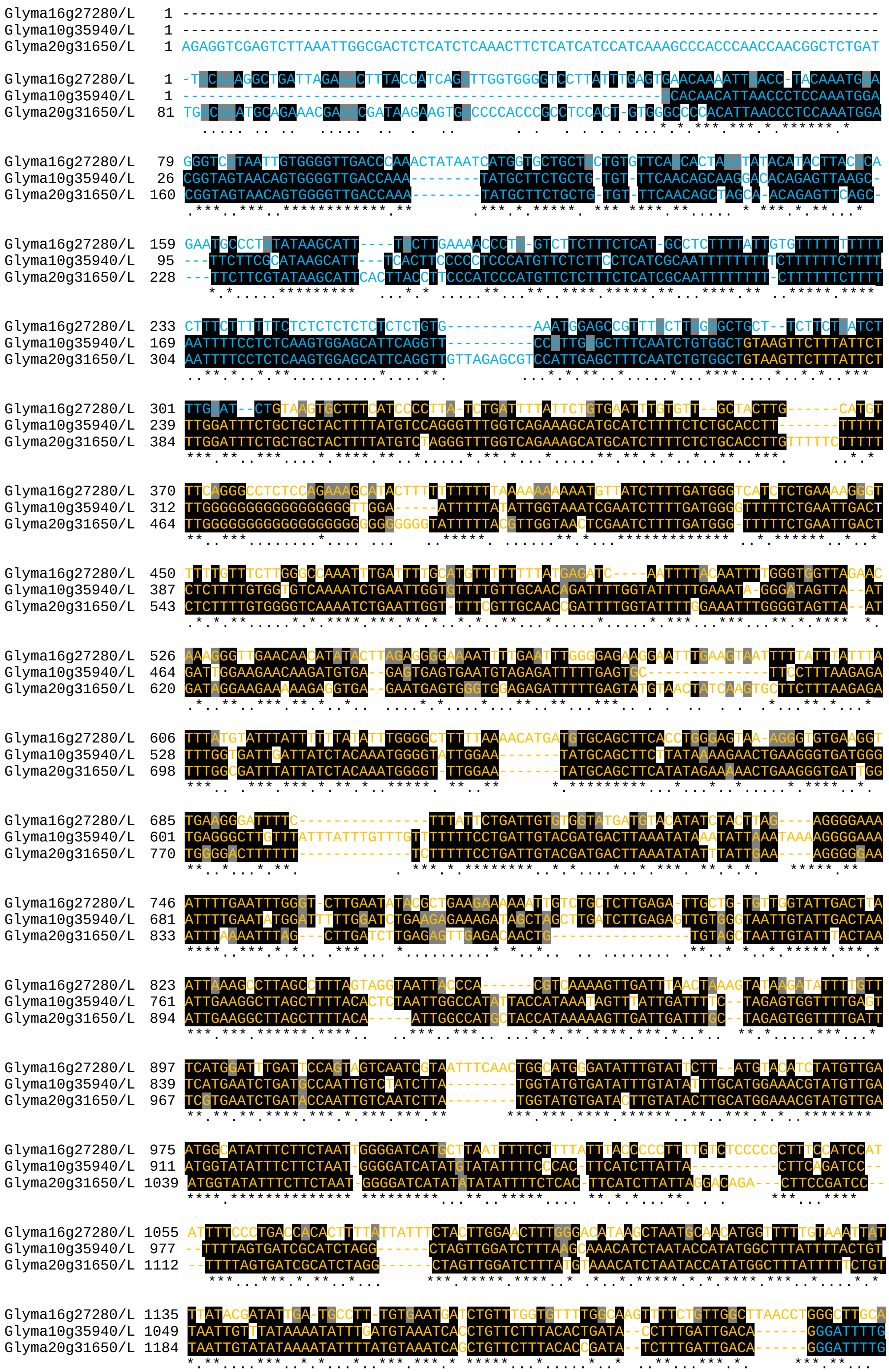
Glyma16g27280/L 1 --
Glyma10g35940/L 1 --
Glyma20g31650/L 1 AGAGGTCGAGTCTTAAATTGGCGACTCTCATCTCAAACTTCTCATCATCCATCAAAGCCCACCCAACCAACGGCTCTGAT
Glyma16g27280/L 1 -TGCAAAGGCTGATTAGAAACTTTACCATCAGATTGGTGGGGTCCTTATTTGAGTGAACAAAATTGACC-TACAAATGAA
Glyma10g35940/L 1 ---ACACAACATTAACCCTCCAAATGGA
Glyma20g31650/L 81 TGACGGATGCAGAAACGAGGCGATAAGAAGTGGCCCCACCCGCCTCCACT-GTGGGCCCCACATTAACCCTCCAAATGGA
Glyma16g27280/L 79 GGGTCATAATTGTGGGGTTGACCCAAACTATAATCATGGTGCTGCTACTGTGTTCAGCACTAGATATACATACTTACACA
Glyma10g35940/L 26 CGGTAGTAACAGTGGGGTTGACCAAA--------TATGCTTCTGCTG-TGT-TTCAACAGCAAGGACACAGAGTTAAGC-
Glyma20g31650/L 160 CGGTAGTAACAGTGGGGTTGACCAAA--------TATGCTTCTGCTG-TGT-TTCAACAGCTAGCA-ACAGAGTTCAGC-
Glyma16g27280/L 159 GAATGCCCTATATAAGCATT----TTCTTGAAAACCCTC-GTCTTCTTTCTCAT-GCCTCTTTTATTGTGTTTTTTTTTT
Glyma10g35940/L 95 ---TTCTTCGCATAAGCATT---TCACTTCCCCCTCCCATGTTCTCTTCCTCATCGCAATTTTTTTTTCTTTTTTCTTTT
Glyma20g31650/L 228 ---TTCTTCGTATAAGCATTCACTTAGCTTCCCATCCCATGTTCTCTTTCTCATCGCAATTTTTTTT-CTTTTTTCTTTT
Glyma16g27280/L 233 CTTTCTTTTTTCTCTCTCTCTCTCTCTGTG----------AAATGGAGCCGTTTGCTTAGAGCTGCT--TCTTCTGATCT
Glyma10g35940/L 169 AATTTTCCTCTCAAGTGGAGCATTCAGGTT----------CCATTGGGCTTTCAATCTGTGGCTGTAAGTTCTTTATTCT
Glyma20g31650/L 304 AATTTTCCTCTCAAGTGGAGCATTCAGGTTGTTAGAGCGTCCATTGAGCTTTCAATCTGTGGCTGTAAGTTCTTTATTCT
Glyma16g27280/L 301 TTGAAT--CTGTAAGTGCTTTCATCCCTTA-TCTGATTTTATTCTGTGAATTTGTGTT--GCTACTTG------CATGT
Glyma10g35940/L 239 TTGGATTTCTGCTGCTACTTTTATGTCCAGGGTTTGGTCAGAAAGCATGCATCTTTTCTCTGCACCTT-------TTTTT
Glyma20g31650/L 384 TTGGATTTCTGCTGCTACTTTTATGTCTAGGGTTTGGTCAGAAAGCATGCATCTTTTCTCTGCACCTTGTTTTTCTTTTT
Glyma16g27280/L 370 TTCAGGGCCTCTCCAGAAAGCATACTTTTTTTTTTTAAAAAAAAATGTTATCTTTTGATGGGTCATCTCTGAAAAGGGT
Glyma10g35940/L 312 TTGGGGGGGGGGGGGGGGTTGGA-----ATTTTTATATTGGTAAATCGAATCTTTTGATGGGGTTTTTCTGAATTGACT
Glyma20g31650/L 464 TTGGGGGGGGGGGGGGGGGGGGGGGGGTATTTTTACGTTGGTAACTCGAATCTTTTGATGGG-TTTTTCTGAATTGACT
Glyma16g27280/L 450 TTTTGTTTCTTGGGCCAAATTTGATTTTGCATGTTTTTTTTATGAGATC----AATTTTACAATTTTGGGTGGTTAGAAC
Glyma10g35940/L 387 CTCTTTTGTGGTGTCAAAATCTGAATTGGTGTTTTGTTGCAACAGATTTTGGTATTTTTGAAATA-GGGATAGTTA--AT
Glyma20g31650/L 543 CTCTTTTGTGGGGTCAAAATCTGAATTGGT-TTTCGTTGCAACCGATTTTGGTATTTTGGAAATTTGGGGTAGTTA--AT
Glyma16g27280/L 526 AAAGGGTTGAACAACATATACTTAGAGGGGAAAATTTTGAATTTGGGGAGAAGGAATTTGAAGTAATTTTTATTTATTTA
Glyma10g35940/L 464 GATTGGAAGAACAAGATGTGA--GAGTGAGTGAATGTAGAGATTTTTGAGTGC--------------TTCCTTTAAGAGA
Glyma20g31650/L 620 GATAGGAAGAAAAAGAGGTGA--GAATGAGTGGGTGGAGAGATTTTTGAGTATGTAACTATCAAGTGCTTCTTTAAGAGA
Glyma16g27280/L 606 TTTATGTATTTATTTTTTATATTTGGGGCTTTTTAAAACATGATGTGCAGCTTCACCTGGGAGTAA-AGGGTGTGAAGGT
Glyma10g35940/L 528 TTTGGTGATTGATTATCTACAAATGGGGTATTGGAA-------TATGCAGCTTCTTATAAAAGAACTGAAGGGTGATGGG
Glyma20g31650/L 698 TTTGGCGATTTATTATCTACAAATGGGGT-TTGGAA-------TATGCAGCTTCATATAGAAAAACTGAAGGGTGATTGG
Glyma16g27280/L 685 TGAAGGGATTTTC---------------TTTATTCTGATTGTGTGGTATGATGTACATATCTACTTAG----AGGGGAAA
Glyma10g35940/L 601 TGAGGGCTTGTTTATTTATTTGTTTGTTTTTTTCCTGATTGTACGATGACTTAAATATAAATATTAAATAAAAGGGGAAA
Glyma20g31650/L 770 TGGGGACTTTTTT-------------TCTTTTTCCTGATTGTACGATGACTTAAATATATATTTATTGAA----AGGGGGAA
Glyma16g27280/L 746 ATTTTGAATTTGGGT-CTTGAATATACGCTGAAGAAAAAATTGTCTGCTCTTGAGA-TTGCTG-TGTTGGTATTGACTTA
Glyma10g35940/L 681 ATTTTGAATATGGATTTTTGGATCTGAAGAGAAAGATAGCTAGCTTGATCTTGAGAGTTGTGGGTAATTGTATTGACTAA
Glyma20g31650/L 833 ATTTAAAATTTAG---CTTGATCTTGAGAGTTGAGACAACTG---------------TGTAGCTAATTGTATTTACTAA
Glyma16g27280/L 823 ATTAAAGCCTTAGCCTTTAGTAGGTAATTACCCA------CGTCAAAAGTTGATTTAACTAAAGTATAAGATATTTTTGTT
Glyma10g35940/L 761 ATTGAAGGCTTAGCTTTTACACTCTAATTGGCCATATTACCATAAATAGTTTATTGATTTTC--TAGAGTGGTTTTGAGT
Glyma20g31650/L 894 ATTGAAGGCTTAGCTTTTACA-----ATTGGCCATGCTACCATAAAAAGTTGATTGATTTGC--TAGAGTGGTTTTGATT
Glyma16g27280/L 897 TCATGGATTTGATTCCAGTAGTCAATCGTAATTTCAACTGGCATGGGATATTTGTATTCTT--ATGTACATCTATGTTGA
Glyma10g35940/L 839 TCATGAATCTGATGCCAATTGTCTATCTTA--------TGGTATGTGATATTTGTATATTTGCATGGAAACGTATGTTGA
Glyma20g31650/L 967 TCGTGAATCTGATACCAATTGTCAATCTTA--------TGGTATGTGATACTTGTATACTTGCATGGAAACGTATGTTGA
Glyma16g27280/L 975 ATGGCATATTTCTTCTAATTGGGGATCATGCTTAATTTTTCTTTTATTTACCCCCTTTTGTCTCCCCCCTTTCCATCCAT
Glyma10g35940/L 911 ATGGTATATTTCTTCTAAT-GGGGATCATATGTATATTTTCCCAC-TTCATCTTATTA----------CTTCAGATCC--
Glyma20g31650/L 1039 ATGGTATATTTCTTCTAAT-GGGGATCATATATATATTTTTCTCAC-TTCATCTTATTAGGACAGA---CTTCCGATCC--
Glyma16g27280/L 1055 ATTTTCCCTGACCACACTTTTATTATTTCTACTTGGAACTTTGGGACATAAGCTAATGCAACATGGTTTTTGTAAATTAT
Glyma10g35940/L 977 --TTTTAGTGATCGCATCTAGG------CTAGTTGGATCTTTAAGCAAACATCTAATACCATATGGCTTTATTTTACTGT
Glyma20g31650/L 1112 --TTTTAGTGATCGCATCTAGG------CTAGTTGGATCTTTATGTAAACATCTAATACCATATGGCTTTATTTTTCTGT
Glyma16g27280/L 1135 TTATACGATATTGA-TGCCTT-TGTGAATGATCTGTTTGGTGTTTTGGCAAGTTTCTGTTGGCTTAACCTGGGCTTGCA
Glyma10g35940/L 1049 TAATTGTTTATAAAATATTTGATGTAAATCACCTGTTCTTTACACTGATA--CCTTTGATTGACA-----GGGATTTTG
Glyma20g31650/L 1184 TAATTGTATATAAAATATTTTATGTAAATCAGCTGTTCTTTACACCGATA--TCTTTGATTGACA-----GGGATTTTG

```
Glyma16g27280/L 1213 AAAGCTTCGTTTTTTGATTAAGCCCAATGATGTTGTACTTTGATTTTCAGTGTTGTTGGGATAAGGCTTTGTTGTTGTTG
Glyma10g35940/L 1121 ATTTCTCTGCGTTTAAAATGGATCCAAAAGGAAGCTT-------------------------------------------
Glyma20g31650/L 1256 ATCTCTCTGAGTTTAAAATGGATCCAAAAGGAAGCTT-------------------------------------------
                     *. .**..* .***..*.*....****........*..*.**

Glyma16g27280/L 1293 TATTTTGTACTATATCATCATGTTCCTTAACTATTTGCCTTTGATTTTCAGTGATCTAGATATCTAGATACAGAATTGGA
Glyma10g35940/L      --------------------------------------------------------------------------------
Glyma20g31650/L      --------------------------------------------------------------------------------


Glyma16g27280/L 1373 AATGGATTCAAATGGGAGCCT
Glyma10g35940/L      ----------------------
Glyma20g31650/L      ----------------------
```

Fig S1: Alignment for 5’-untranslated regions (5’ UTR) of *GmSTOP1s*. The coding sequence, 5’ UTR and introns in the 5’ UTR are presented in red, blue and orange colored letters, respectively. Asterisks (*) and dots (.) indicate the amino acids that are conserved in all three or only two *GmSTOP1* genes, respectively.
