# Supplementary material for: Functional Conservation and Divergence of Soybean GmSTOP1 Members in Proton and Aluminum Tolerance
Source: Front Plant Sci. 2018 Apr 26;9:570. doi: 10.3389/fpls.2018.00570 (PMC5932199; doi:10.3389/fpls.2018.00570)
Supplement: Supplementary file 3 [file Image_2.PDF]

Fig S2.

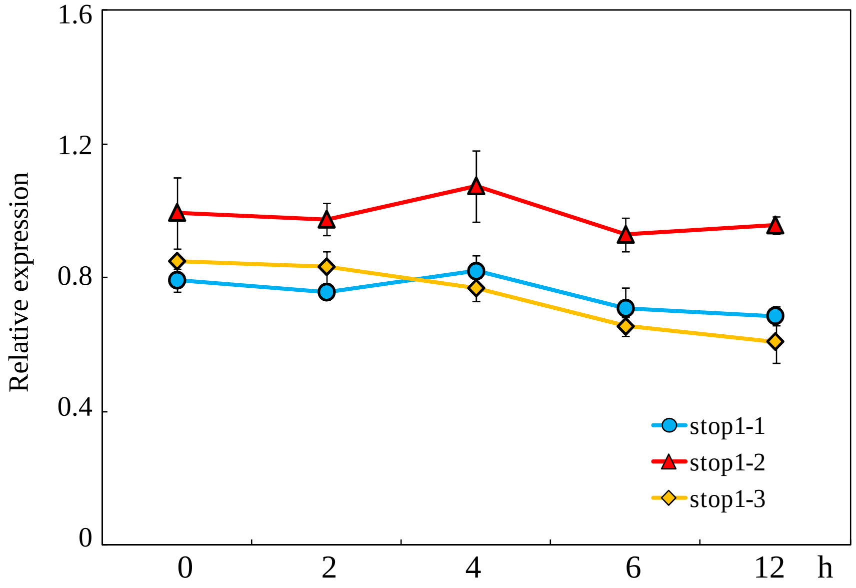

Figure S2. The expression of *GmSTOP1*s in response to low pH stress. The relative expression of the three *GmSTOP1*s identified in soybean was investigated in soybean roots apexes (0-2 cm) in response to low pH treatment (pH 4.2) for 0, 2, 4, 6, or 12 hours.
