# Supplementary material for: Functional Conservation and Divergence of Soybean GmSTOP1 Members in Proton and Aluminum Tolerance
Source: Front Plant Sci. 2018 Apr 26;9:570. doi: 10.3389/fpls.2018.00570 (PMC5932199; doi:10.3389/fpls.2018.00570)
Supplement: Supplementary file 4 [file Image_3.PDF]

Fig S3.

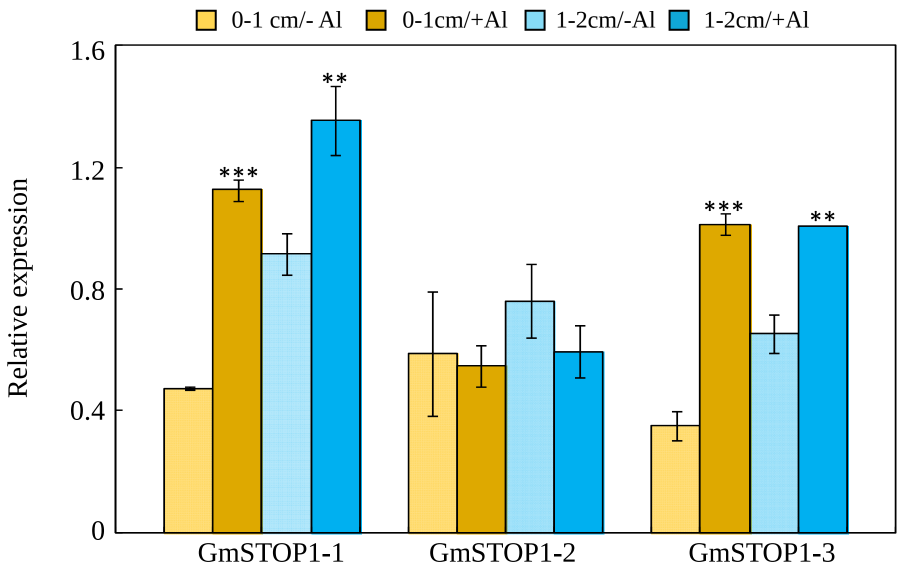

Figure S3. The expression of *GmSTOP1*s in root tips responsive to Al stress. The relative expression of the three *GmSTOP1*s identified in soybean was investigated in soybean roots apexes (0-1cm and 1-2 cm from root tips) subjected to Al treatments (pH 4.2) for 4 hours. (**: $0.001<P<0.01$; ***: $P<0.001$)
