# Supplementary material for: Functional Conservation and Divergence of Soybean GmSTOP1 Members in Proton and Aluminum Tolerance
Source: Front Plant Sci. 2018 Apr 26;9:570. doi: 10.3389/fpls.2018.00570 (PMC5932199; doi:10.3389/fpls.2018.00570)
Supplement: Supplementary file 5 [file Image_4.PDF]

Fig S4.

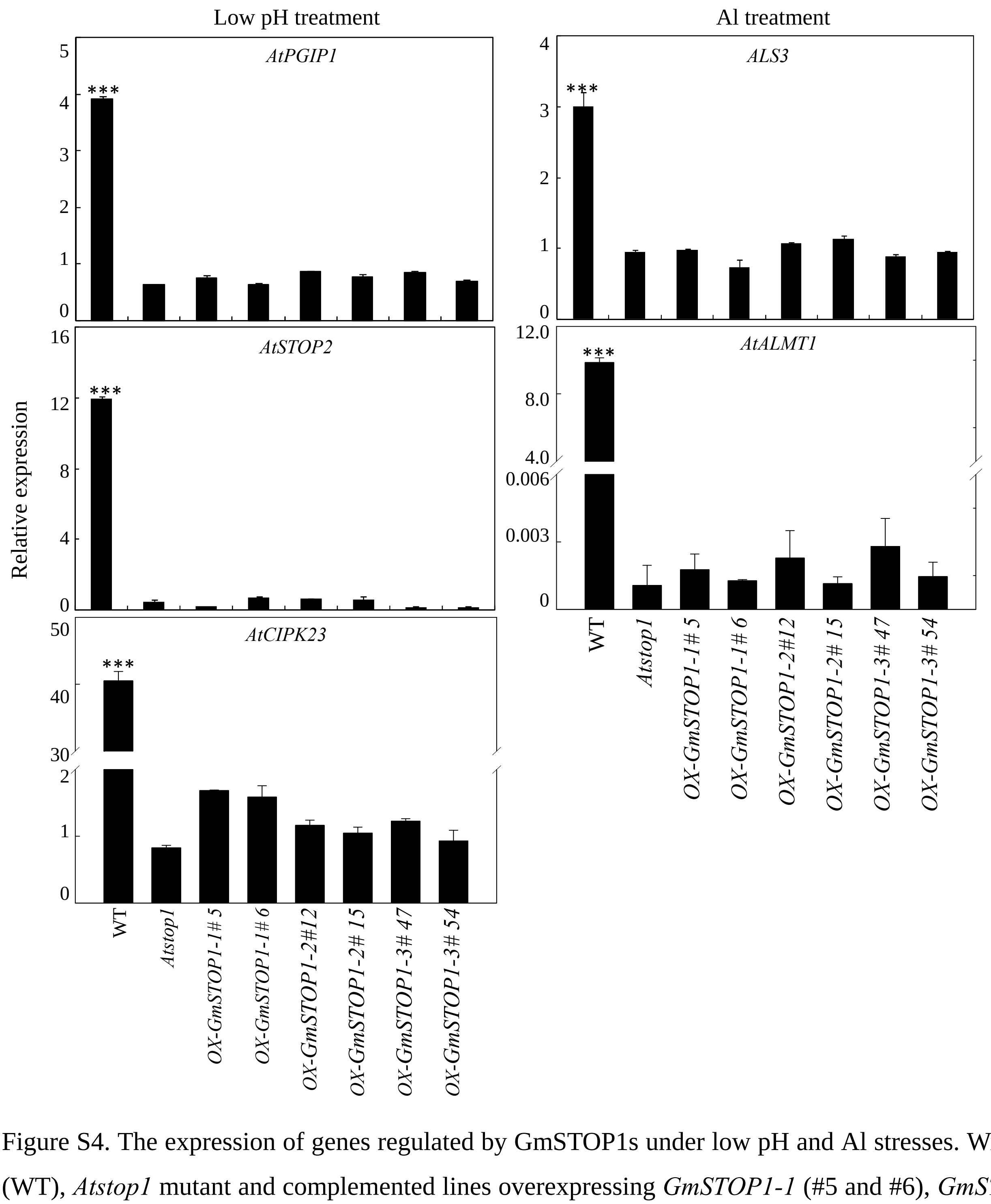

Figure S4. The expression of genes regulated by GmSTOP1s under low pH and Al stresses. Wild-type (WT), *Atstop1* mutant and complemented lines overexpressing *GmSTOP1-1* (#5 and #6), *GmSTOP1-2* (#12 and #15), and *GmSTOP1-3* (#47 and #54) were exposed to low pH (pH 4.7) and Al treatments ($AlCl_3$: 2 μM; pH 5.0) for 24 h. The expression of *PGIP1*, *STOP2* and *CIPK23* was quantified in plants grown in the low pH treatment, while expression of *ALS3* and *AtALMT1* was quantified in plants grown in the Al treatment. UBQ1 transcript levels were used as the internal standard. Data are expressed as means of four replicates. Asterisks indicate significant differences in comparison to the *Atstop1* mutant (***: P<0.001).
